# Dominant Patterns of Information Flow in the Propagation of the Neuromagnetic Somatosensory Steady-State Response

**DOI:** 10.3389/fncir.2018.00118

**Published:** 2019-01-15

**Authors:** Vasily A. Vakorin, Bernhard Ross, Sam M. Doesburg, Urs Ribary, Anthony R. McIntosh

**Affiliations:** ^1^Department of Biomedical Physiology and Kinesiology, Simon Fraser University, Burnaby, BC, Canada; ^2^Behavioral and Cognitive Neuroscience Institute, Simon Fraser University, Burnaby, BC, Canada; ^3^Rotman Research Institute, Baycrest Centre for Geriatric Care, Toronto, ON, Canada; ^4^Department of Psychology, Simon Fraser University, Burnaby, BC, Canada; ^5^Department of Pediatrics and Psychiatry, University of British Columbia, Vancouver, BC, Canada; ^6^Department of Psychology, University of Toronto, Toronto, ON, Canada

**Keywords:** magnetoencephalography (MEG), somatosensory system, steady-state response, causality analysis, complexity analysis

## Abstract

Methods of functional connectivity are applied ubiquitously in studies involving non-invasive whole-brain signals, but may be not optimal for exploring the propagation of the steady-state responses, which are strong oscillatory patterns of neurodynamics evoked by periodic stimulation. In our study, we explore a functional network underlying the somatosensory steady-state response using methods of effective connectivity. Human magnetoencephalographic (MEG) data were collected in 10 young healthy adults during 23-Hz vibro-tactile stimulation of the right hand index finger. The whole-brain dynamics of MEG source activity was reconstructed with a linearly-constrained minimum variance beamformer. We applied information-theoretic tools to quantify asymmetries in information flows between primary somatosensory area SI and the rest of the brain. Our analysis identified a pattern of coupling, leading from area SI to a source in the secondary somato-sensory area SII, thalamus, and motor cortex all contralateral to stimuli as well as to a source in the cerebellum ipsilateral to the stimuli. Our results support previously reported empirical evidence collected both in *in vitro* and *in vivo*, indicating critical areas of activation of the somatosensory system at the level of systems neuroscience.

## Introduction

Electrophysiological steady-state responses (SSR) entrained to the frequency and phase of periodic stimuli provide a non-invasive method to test the integrity of sensory pathways. Many studies characterized the shapes of individual SSR and their dominant frequencies, but mapping of the SSR as the propagation of a neural signal within the wider network underlying somatosensory function remains largely unexplored. A combination of steady-state responses and statistical tools explicitly describing information transfers could provide a data-informed approach for studying the integrity of sensory pathways at the level of systems neuroscience.

In general, the steady-state response arises from strong oscillatory dynamics of neural activity evoked by periodic stimulation, in contrast to temporal patterns of the transient changes in signal dynamics caused by the onsets of stimuli, such as event-related potentials (ERPs). In our study, we will focus on the somatosensory steady-state response (SSSR), which is elicited by periodic tactile stimulation, and provides effective means to investigate oscillatory brain sensory networks (Nangini et al., 2006). The SSSR is typically observed at the frequencies of the driving stimulus, possibly with higher harmonics, reaching a resonance-like maximum in amplitude of around 20Hz (Snyder, 1992). The steady-state response is believed to be a result of the interplay between the on-going activity and afferent input, reflecting the intrinsic dynamic properties of the sensory networks at the scale of neuronal populations (Engel et al., 2001).

Previous studies have extensively linked the somatosensory steady-state response with primary somatosensory area SI using the model of a single equivalent current dipole under the assumption that response to tactile stimuli can be localized only to one specific location (Baumgartner et al., 1993; Hari et al., 1993). Multiple dipole modeling of electric brain sources is possible but requires more complex assumptions about the number of underlying sources, their locations and possible orientations of the dipoles (Hoshiyama et al., 1997). In contrast, model-free beamformer techniques can be used in an attempt to reconstruct whole-head volumetric maps of neural dynamics (Robinson and Rose, 1992; Van Veen et al., 1997). In both cases, dipole modeling or beamforming, not much research has been performed to study the propagation of the steady-state response throughout the wider network underlying human somatosensory function.

Typical studies on steady-state responses characterize source activation based on similarity between source dynamics (functional connectivity), which is ultimately driven by their similarity with stimuli (Bardouille and Ross, 2008; Schlee et al., 2008). In an extreme case, two perfect sine waves can be considered as highly correlated, but from the point of information-theoretic analysis, such a model assumes no information flow between them. In contrast to functional connectivity, effective connectivity (an influence which one signal exerts over another signal) essentially quantifies information transfer, thus being an attempt to explicitly describe information processing in a network (Vicente et al., 2011). Typical directionality measures are asymmetric, and can be applied in both directions, estimating an amount of information transferred from one source to another, and vice versa. Asymmetry in informant transfer would then indicate the dominant directionality in propagation of information in the network.

An analysis of dominant information flow with respect to SI would provide more insight in reconstructing the propagation of somatosensory response, inferring the directionality of communications within the network of distributed neuronal ensembles. In this study, we establish a fully data-driven approach using information-theoretic tools to describe the effective connectivity between neuromagnetic sources activated in response to the mechanic pressure periodically applied to a finger. We explore the exchange of information between the primary somatosensory area SI and the rest of the brain. We identify several distinctive sources which are localized contralaterally to stimuli in the secondary somatosensory area SII, thalamus, motor cortex, and ipsilaterally in the cerebellum. Specifically, we show that more information is transferred from SI to all those sources, than in the reverse direction.

## Materials and Methods

### Experiment

MEG data were collected at a sample rate of 1,250 Hz with a bandwidth of 0–300 Hz using a 151-channel whole head first-order gradiometer system (CTF, Coquitlam, BC, Canada). Subjects (*n* = 10) were seated upright with their head resting in the helmet shaped scanner. Head localization coils were placed on the nasion, and left and right pre-auricular points for co-registration of MEG data with subject-specific anatomical MR images. A small elastic air bladder was fitted to the right index finger pad. Vibrotactile stimulation was applied to the finger by pressurizing the bladder with 23 Hz trains of 10 ms compressed air pulses of 3 s in duration. The inter-train interval was randomly distributed between 3 and 5 s. A white noise masking sound was binaurally presented at 90 dB SPL via insert phones to mask sounds possibly associated with the stimulator. MEG and the driving signal for the stimulator were collected simultaneously for 10 min. Subjects watched a subtitled silent movie and were asked to stay alert during the MEG recording. No responses were required. Informed and written consent was obtained from each subject before participating in the study, which had been approved by the Research Ethics Board at Baycrest Centre for Geriatric Care. More details on the data acquisition can be found in Bardouille and Ross (2008).

### Asymmetry in Information Transfer

In this section we describe a non-linear method to quantify the transfer of information between two processes. This method explicitly assumes that sensory processing may be considered the result of generation and transformation of cooperative modes of neural activity (Bressler, 1995, 2002; McIntosh, 1999). Specifically, the principles emphasize the integrative capacity of the brain in terms of ensembles of coupled neural systems interacting in a non-linear way (Nunez, 1995; Jirsa and McIntosh, 2007). Typically, in a non-linear analysis of EEG or MEG, it is assumed that individual time series, *x*_*t*_ and *y*_*t*_, represent the manifestation of underlying multi-dimensional non-linear dynamics (Stam, 2005). To estimate the exchange of information between two systems, we reconstruct, from time series of observations, the dynamics in the multi-dimensional state space of the underlying model. It can be done with time delay embedding

(1) $$\begin{array}{l} x_{t}={\left(x_{t},x_{t-\tau_{x}},\ldots ,x_{t-\tau_{x}\left(d_{x}-1\right)}\right)}^{T} \end{array}$$

(2) $$\begin{array}{l} y_{t}={\left(y_{t},y_{t-\tau_{y}},\ldots ,y_{t-\tau_{y}\left(d_{y}-1\right)}\right)}^{T} \end{array}$$

wherein each time series, *x*_*t*_ and *y*_*t*_, is converted to a sequence of vectors in a multi-dimensional space. Here *d*_*x*_ and *d*_*y*_ are embedding dimensions, and τ_*x*_ and τ_*y*_ are embedding delays measured in multiples of the sampling interval. Note that the ultimate goal is not to reconstruct an orbit in the state space that is closest to the true one. However, Takens' embedding theorem states that if the embedding dimension *m* is sufficiently high, the macrocharacteristics of a dynamic system such as the entropy or information content of a signal can be approximated (Takens, 1981).

Information transfer from system *x*_*t*_ to *y*_*t*_ can be quantified using information-theoretic tools. Intuitively, *y*_*t*_ is a nonlinear cause of *x*_*t*_, denoted as *Y* → *X*, if past and present values of *y*_*t*_ contain information about future values of *x*_*t*_, provided that information about past and present of *x*_*t*_ itself is excluded (Palus et al., 2001). The transfer of information from *Y* to *X* is quantified as the conditional mutual information *I*(*x*_*t* + *k*_, ***y***_*t*_ | ***x***_*t*_) between *x*_*t* + *k*_ and ***y***_*t*_ given ***x***_*t*_. Palus and Vejmelka (2007) showed that under certain conditions, *I*(*x*_*t* + *k*_, ***y***_*t*_ | ***x***_*t*_) is equivalent to the measure of transfer entropy *T*(*Y* → *X*) proposed by Schreiber (2000). The transfer of information *T*(*Y* → *X*) or *I*(*x*_*t* + *k*_, ***y***_*t*_ | ***x***_*t*_) can be expressed in terms of individual *H*(·) and joint entropies *H*(·, ·) and *H*(·, ·, ·) as following:

(3) $$\begin{array}{l} T_{k}\left(Y\to X\right)\equiv I\left(x_{t+k},y_{t}|x_{t}\right)=H\left(x_{t+k},x_{t}\right)+H\left(y_{t},x_{t}\right)\\ \;-H\left(x_{t+k},y_{t},x_{t}\right)-H\left(x_{t+k}\right) \end{array}$$

where the index *k* is used to designate dependence of the transfer information on the future lag *k*, which is measured in units of data points. In a similar way we define the transfer of information *T*_*k*_(*X* → *Y*) from *X* to *Y*:

(4) $$\begin{array}{l} T_{k}\left(X\to Y\right)\equiv I\left(y_{t+k},x_{t}|y_{t}\right) \end{array}$$

In general, it holds that *I*(*y*_*t* + *k*_, ***x***_*t*_ | ***y***_*t*_) ≠ *I*(*x*_*t* + *k*_, ***y***_*t*_ | ***x***_*t*_). The difference between the two measures, *T*_*k*_(*X* → *Y*) and *T*_*k*_(*Y* → *X*), characterizes an asymmetry in the predictive power between two signals, and thus indicate the directionality of the dominant information transfer between the systems underlying the observed signals. Specifically, we define the asymmetry in information transfer *D*_*k*_(*Y* → *X*) as:

(5) $$\begin{array}{l} D_{k}\left(Y\to X\right)=T_{k}\left(Y\to X\right)-T_{k}\left(X\to Y\right) \end{array}$$

Note that this statistic is anti-symmetrical, *e.i*. *D*_*k*_(*Y* → *X*) = −*D*_*k*_(*X* → *Y*), and produces zero values between identical signals.

### Estimation of Information Transfer

Until now we described the theoretical aspects of constructing a measure of the information transfer (3) and (4). A crucial issue is how to estimate them from finite noisy time series. Here we give a short description of the pipeline which was tested in previous studies using linear and linear models (Chávez et al., 2003; Vakorin et al., 2009, 2012, 2013).

There are three points to note. First, in estimating the measure (5), to reduce the variance of estimation error and to increase the robustness of the results, *T*_*k*_(*Y* → *X*) and *T*_*k*_(*X* → *Y*) can be substituted by their averaged estimates, < *T*_*k*_(*Y* → *X*) >_*k*_ and < *T*_*k*_(*X* → *Y*) >_*k*_, respectively, where < … >_*k*_ denotes averaging over a selected range of future lags *k* = 1, …, *k*_*max*_ (Palus et al., 2001). Thus, the measure *D*(*Y* → *X*) can be expressed as

(6) $$\begin{array}{l} D\left(Y\to X\right)=<T_{k}\left(Y\to X\right){>}_{k}-<T_{k}\left(X\to Y\right){>}_{k} \end{array}$$

The second issue is estimation of the entropies themselves. The straightforward approach is to divide the state-space into bins, *i* = 1, 2, 3, …, of some size δ and calculate the entropy of the multidimensional dynamics through constructing a multidimensional histogram, estimating probabilities of being in the bin *i*, *p*_*i*_(***x***, δ). This study took another approach, as proposed by Prichard and Theiler (1995) who estimated individual and joint entropies *H*(***x***) through the corresponding correlation integral *C*_*q*_(***x**, r*).

Correlation integral is interpreted as the likelihood that the distance between two randomly chosen points or embedding vectors, ***x***_*i*_ and ***x***_*j*_, representing the multi-dimensional dynamics of ***x*** at times *i* and *j*, is smaller than some characteristic scale length *r*. The entropy can be estimated through correlation integral as

(7) $$\begin{array}{l} H\left(x\right)=-\underset{i\left(\text{bins}\right)}{\sum }p_{i}\left(x,\delta \right)\log_{2}p_{i}\left(x,\delta \right)\approx -\log_{2}C_{q}\left(x,r\right) \end{array}$$

where the correlation integral *C*_*q*_(***x**, r*) is a function of the scale parameter *r*, which in general can be related to the bin size δ. The parameter *q* is the integral order.

In turn, the *C*_*q*_(***x**, r*) is defined as

(8) $$\begin{array}{l} C_{q}\left(x,r\right)=\frac{1}{N{\left(N-1\right)}^{q-1}}\times \overset{N}{\underset{j=1}{\sum }}\left[\underset{i\neq j}{\sum }\Theta \left(r-∥x_{i}-x_{j}∥\right)\right] \end{array}$$

where *N* is the number of data points (embedding vectors), Θ is the Heaviside function, and ∥·∥ stands for the maximum norm distance between two embedding vectors ***x***_*i*_ and ***x***_*j*_. For a given vector ***x***_*i*_, the function $\underset{i\neq j}{\sum }\Theta \left(r-∥x_{i}-x_{j}∥\right)$ represents the number of the points *j* such that the distance between the *d*-dimensional vectors ***x***_*i*_ and ***x***_*j*_ is less than *r*. In this study, we used the second order (*q* = 2) correlation integral, following Pompe (1993) who proposed to call this a measure of generalized mutual information.

The third point is related to the issue of choosing the optimal embedding parameters, τ and *d*. This is a crucial and, in general, non-trivial step. There are many competing approaches proposed in the literature, and all of them are heuristic and somewhat mutually exclusive. The task can be simplified in case of frequency priors when we are interested in interactions for a specific frequency band (Montez et al., 2006). The idea is that the embedding lag τ should be short enough to capture the fastest oscillations cut off by the highest frequency *f*_*H*_, while the lowest frequency *f*_*L*_ determines the embedding window *d* ×(τ − 1) of a delay vector. The embedding window should be long enough to allow for the slowest processes. This leads to the following estimators:

$$\begin{array}{l} \tau =\frac{f_{s}}{\epsilon f_{H}} \end{array}$$

(9) $$\begin{array}{l} d=\frac{\epsilon f_{H}}{f_{L}}+1 \end{array}$$

where *f*_*s*_ is the sampling frequency, and ϵ = 2…3 is a constant factor related to the Nyquist sampling theorem, indicating that a process must be sampled at least twice the highest frequency of its fluctuations.

### Workflow of the Analysis

First, MEG data were down-sampled to a 250Hz sampling rate, and separated into trials of five second durations including 1 s pre-stimulus and 1 s post-stimulus intervals. The stimuli were of 3 s durations. For each MEG sensor and each trial, the baseline correction was achieved by subtracting the mean signal strength across the pre-stimulus interval, and a band pass filter was applied between 16 and 28 Hz. Based on the prior knowledge about the SSSR, we isolated the steady-state MEG data as the time interval from 500ms after stimulus onset to the end of the stimulus train, [0.5s 3.0s]. The first half second was discarded to avoid possible transitory effects.

Source activity was reconstructed with synthetic aperture magnetometry (SAM) (Robinson and Vrba, 1999). The nasion and left/right pre-auricular points were identified on the subjects' anatomical MRI to co-register the MEG data. Isolating the scalp in the MRI generated a realistic head model for source estimation. Based on this head model and the steady-state MEG data, a weighting coefficient set was determined at each node on a 5 × 5 × 5 mm grid encompassing the whole brain using a data-driven linearly constrained minimum variance beamformer (Robinson and Rose, 1992; Van Veen et al., 1997). The coefficient sets, in linear combination with the MEG data, estimated the source activity at each grid node to generate whole brain estimates of neuronal activity over time. For more details on the preprocessing procedures and source reconstruction, see Bardouille and Ross (2008). The methods for estimating asymmetries in information transfer were applied to the reconstructed steady-state source activity.

Further, the steady-state time series for each voxel were normalized to have the mean of zero and the variance of one. Then, the delay vectors was constructed with the embedding dimension of 5 and the lag of 4 time points according to the criteria (9), assuming that *f*_*L*_ = 16Hz and *f*_*H*_ = 28 Hz. In a previous study (Vakorin et al., 2010), for each subject, we identified a source localized to the primary somatosensory area SI, contralateral to stimuli. This source was characterized as having higher signal regularity among other sources (MEG virtual channels), based on the analysis of sample entropy (Vakorin et al., 2010). Net information transfer as defined in (6) was calculated between area SI and all other virtual channels, for all the single trials, with subsequent averaging across trials.

The volumetric maps of the net information transfer with respect to area SI were created and co-registered with the anatomical MRI identifying the head localization coil locations in the MR image. Each subject's MRI was transformed into Talairach space, and we applied the same transformation to the maps of information exchange on a subject-by-subject basis. Then, for each virtual channel, one-sample *t*-test was apply to compare the mean score to zero across subjects, and the volumetric maps of *t*-test values and corresponding *p*-values were created. AFNI software (Cox, 1996) was used to visualize the volumetric data, with the *t*-test map superimposed on the subject-averaged anatomical MRI. For each cluster of virtual channels, we computed the mean effect size, averaged across all virtual channels in the given cluster. Effect size was calculated as Hedges' *g* statistic (Hedges, 1981), which was proposed to correct a bias for Cohen's *d* statistic for small sample size (*n* < 20).

## Results

**Figure 1:**
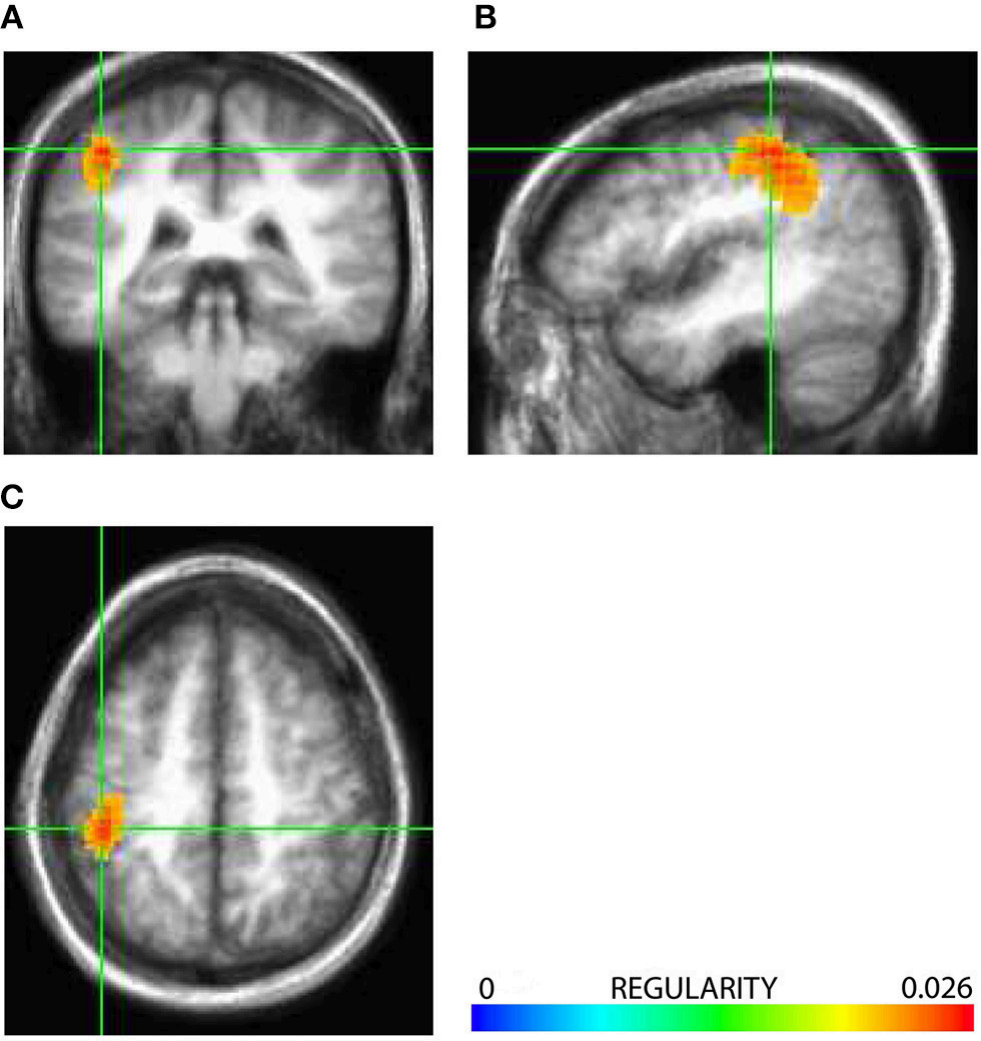
Axial **(A)**, coronal **(B)**, and saggital **(C)** views of volumetric regularity (inverted complexity) map of the somatosensory steady-state response, shown for a peak located in the primary somatosensory area SI. The map is thresholded at the level of 90% of the maximum regularity value of 0.026. Higher values coded with red colors are associated with higher signal regularity of the steady-state response at a given location, compared to other sources.

Previously, having applied an analysis of signal regularity quantified as inverted signal complexity (sample entropy), we localized activation of a source in the somatosensory area SI contralateral to stimuli (Vakorin et al., 2010). Specifically, Figure 1 shows the volumetric subject-averaged activation map for a local peak in area SI. The activation map was thresholded at the level of 90% of its maximum, and was superimposed on the subject-averaged anatomical MRI. The color bar in Figure 1 indicates the magnitude distribution of the measure of signal regularity averaged across subjects. Higher values coded in red colors are associated with an increase in signal regularity of the steady-state response at a given source location. This was a result obtained in our previous study. In the current study, we used the source associated with the subject-specific peak in regularity in area SI, as the seed for an analysis of directionality of coupling from SI to the rest of the brain.

**Figure 2:**
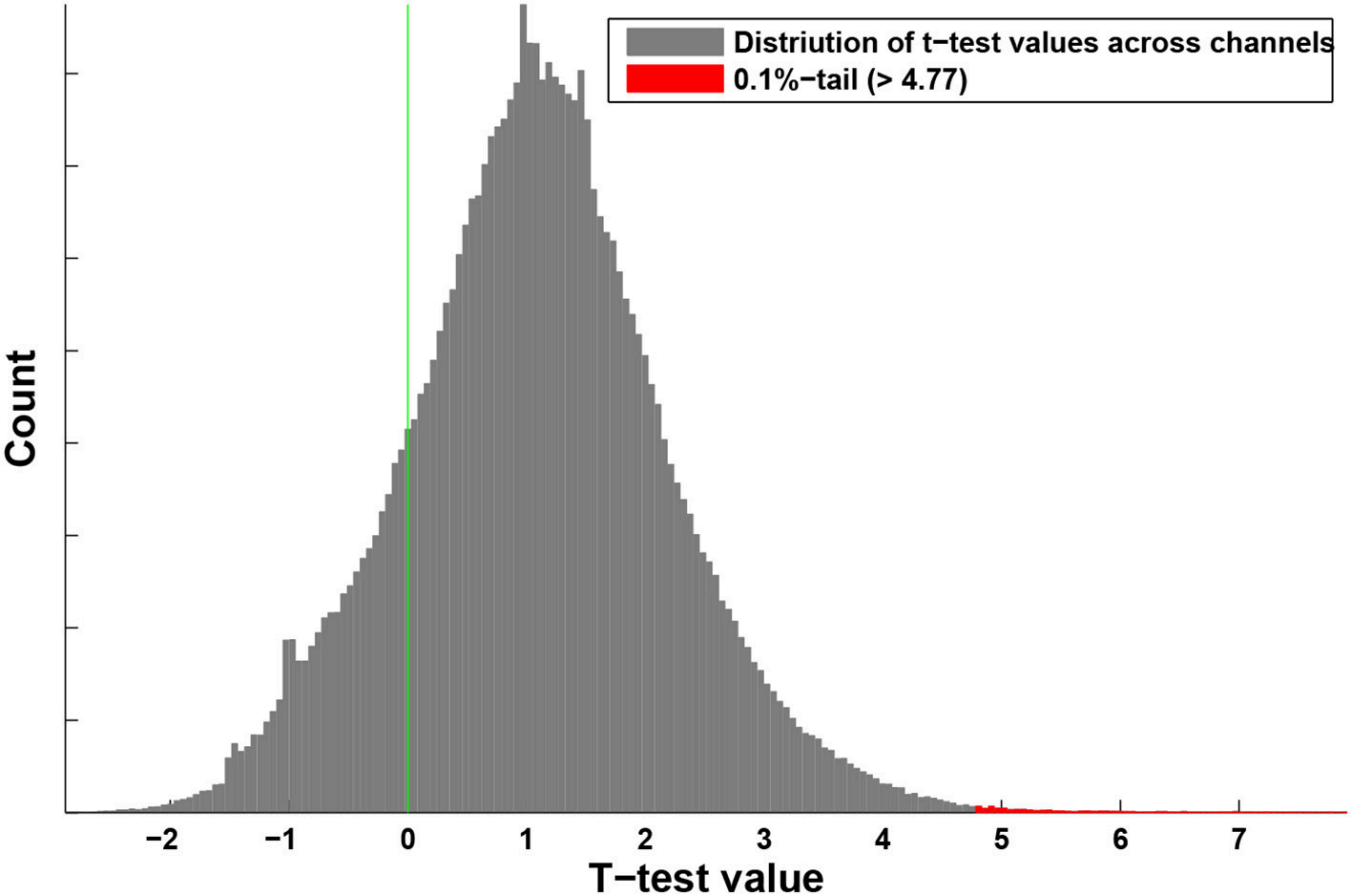
Distribution of the *t*-test values across the virtual channels (voxels). The channels from the upper 0.1%-tail, which is defined by 0.999-quantile = 4.77, represent two brain areas, shown in Figure 3.

For each subject, we generated volumetric maps of the net information transfer with respect to a source in area SI (the seed), with the rest of the brain being the target. For almost all virtual channels, the hypothesis on the normality of the distribution of net information transfer across subjects could not be rejected. The robustness of the asymmetry in information transfer across subjects was then tested with *t*-tests. The distribution of *t*-test values across all the virtual channels is shown in Figure 2. Note that positive values for the t-statistic are associated with the dominant transfer of information from the primary somatosensory area SI, whereas the negative values indicate that net information is coming into area SI. As can be seen from Figure 2, the distribution is skewed toward the positive values, indicating that, on average, area SI is considered a generator of information in the propagation of the somatosensory steady-state response.

**Figure 3:**
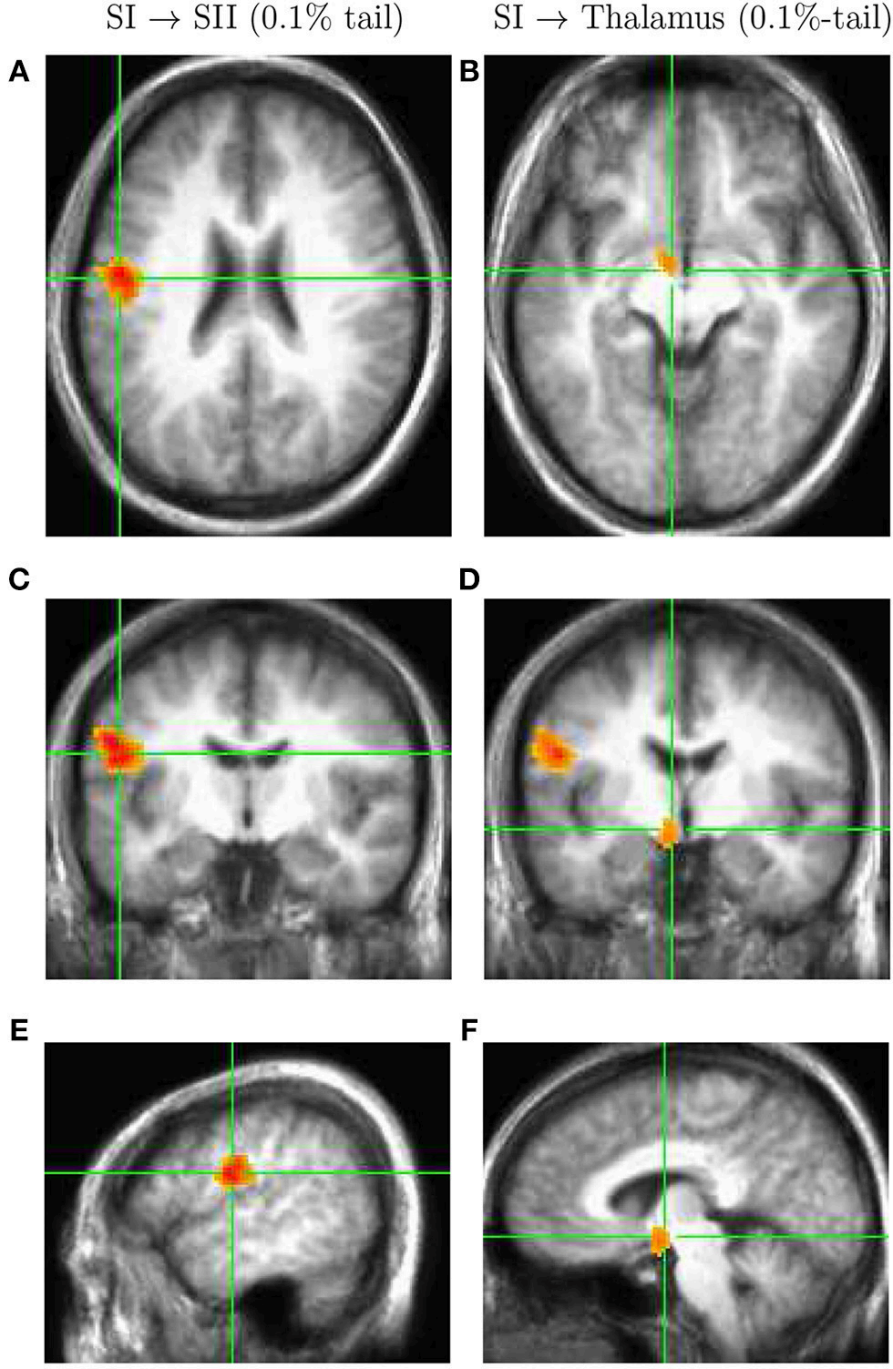
Volumetric map of net information transfer with respect to the primary somatosensory area SI. The map is thresholded at the 0.999-quantile (0.1% tail in Figure 2), and is superimposed on subject-averaged anatomical MRI. Two clusters of virtual channels are identified: secondary somatosensory area SII (left column, **A,C,E**), and a thalamic source (right column, **B,D,F**). The directionality of dominant information transfer is from SI to SII and thalamus.

Next, we identified the upper 0.1%-tail of this distribution defined by the 0.999-quantile equal to 4.77. Then, we used a clustering procedure 3Dclus from AFNI software to find clusters of active voxels, removing clusters below the size limit of 100–200 voxels (the results were robust with respect to the cluster size limit). The two clusters were localized in the secondary somatosensory area SII (Hedges' *g* = 1.4 ± 0.3) and thalamus (Hedges' *g* = 1.3 ± 0.2). Specified in Talairach coordinate system, the peak located in area SII was found to be around x = –53 mm [L], y = –9 mm [P], and z = 27 mm [S], whereas the coordinates of the thalamic source were around x = –6 mm [L], y = –4 mm [P], and z = –4 mm [I]. Figure 3 shows the axial, coronal, and sagittal views of the volumetric map of net information transfer with respect to area SI for these two locations: sub-panels in the left are associated with area SII (Figures 3A,C,E), where sub-panels in the right column illustrate activation in thalamus (Figures 3B,D,G). The map was thresholded at the 0.999-quantile, and was superimposed on the subject-averaged anatomical MRI. Note that red color represents the net transfer of information directed from SI to SII and thalamus.

**Figure 4:**
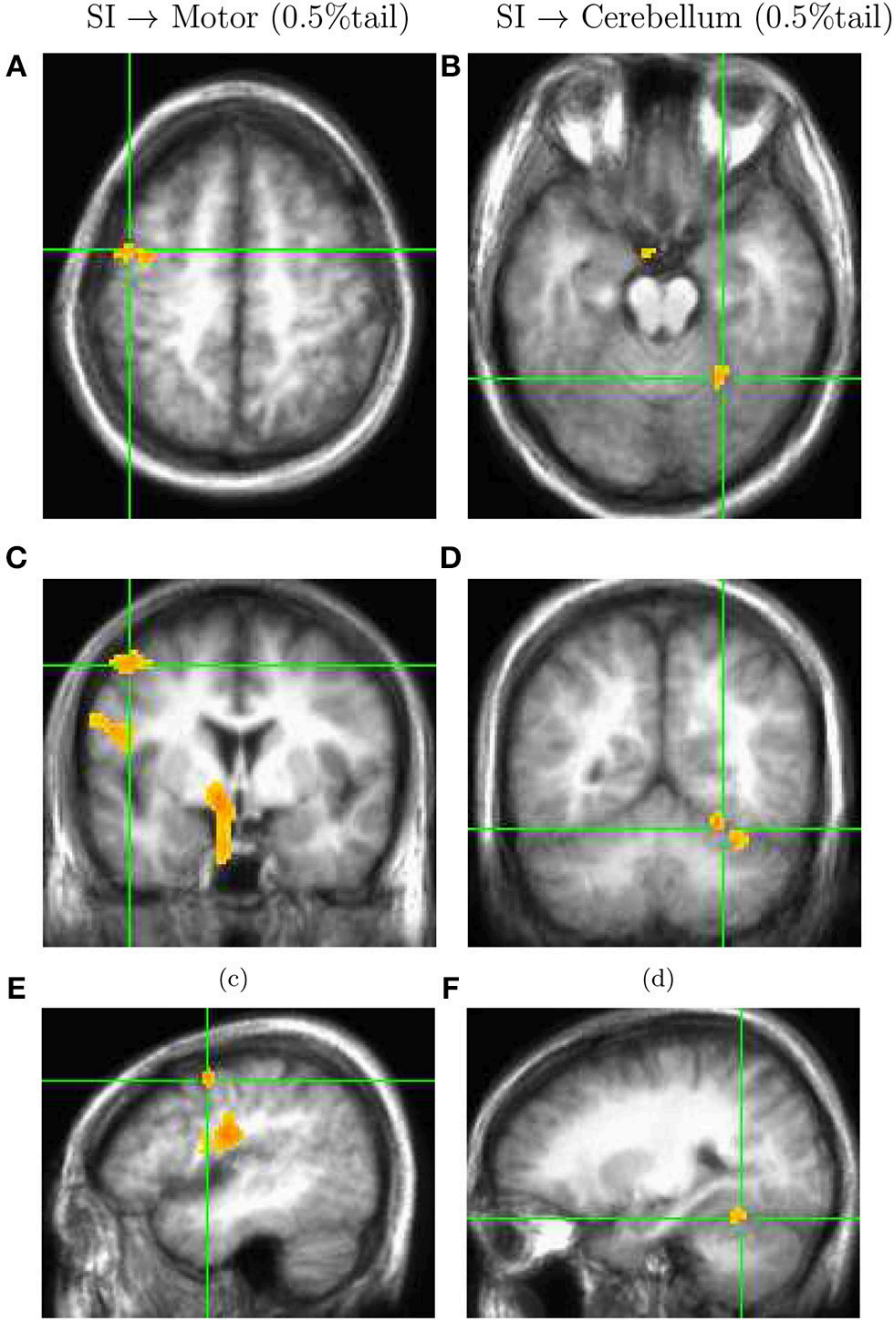
Volumetric map of net information transfer with respect to the primary somatosensory area SI, similar to Figure 3, but with a decreased threshold associated with the 0.5% tail of the distribution in Figure 2. The directionality map is superimposed on subject-averaged anatomical MRI. With the decreased threshold, two more clusters of virtual channels are identified: one in the motor cortex **(A,C,E)**, and one in the cerebellum **(B,D,F)**. The directionality of dominant information transfer is from SI to motor cortex and cerebellum.

With a decreased threshold for the *t*-test statistics associated with a 0.5% tail, similar procedure identified four clusters. Specifically, in addition to sources in area SII and thalamus contralateral to stimuli, we found a source in the motor cortex (Figures 4A,C,E) around x = –44 mm [L], y = –3 mm [P], and z = 54 mm [S] contralaterally, and in the cerebellum ipsilaterally (Figures 4B,D,F) around x = 23 mm [R], y = –53 mm [P], and z = -17 mm [I]. Effect size for both these sources was estimated to be around Hedges' *g* = 1.3 ± 0.1.

## Discussion

Typically, measures for quantifying similarities between the signals are symmetric. Basically, almost all measures of functional connectivity belong to this class. In contrast, asymmetry in the time courses between brain areas may indicate another aspect of coordinated activity. Specifically, a significant difference in the amount of information sent in one direction and in the other, may be indicative of elevated communication between nodes in a functional network. Information generated by a dynamic system (which can be quantified as signal complexity of individual nodes) and information transfer between them would be considered two complementary sides of the interplay between specialization and integration processes (Mišić et al., 2011; Vakorin et al., 2012). An analysis of net information transfer thus would provide more insight in reconstructing the sequence and directionality of interactions occurring in the network of distributed neuronal ensembles, which may be hidden for measures of functional connectivity.

The data used in our study have been differently analyzed in two previous studies (Bardouille and Ross, 2008; Vakorin et al., 2010). Bardouille and Ross (2008) applied a measure called inter-trial coherence (ITC) (Stapells et al., 1987) to identify brain areas that activate synchronously during the steady-state response with consistent phase relations to the vibrotactile stimulation of the right index finger. Although they were able to identify a handful of sensorimotor areas, only area SI activated contralaterally to the stimulus was consistently expressed across all the subjects.

Using the same data, Vakorin et al. (2010) further studied the activation of the somatosensory steady-state response (SSSR) with entropy-based statistics constructed from a combination of information theory and non-linear dynamics. Instead of estimating the consistency of the frequency-specific phase dynamics across trials, this study explored the signal complexity (or regularity) of individual trials, using sample-entropy, a measure that is closely related to the mean rate of information generated by a non-linear system underlying the observed time series (Richman and Moorman, 2000; Vakorin and McIntosh, 2012). In contrast to ITC, this analysis identified activity not only in area SI contralateral to stimuli, but also bilaterally in the PPC, as regions with increased signal regularity, consistently expressed across the subjects.

In the same data, a seed analysis with respect to a source with a higher signal regularity, located in in SI, a measure of pattern synchronization called cross-sample entropy was used to generate synchrony maps between SI and the rest of the brain (Vakorin et al., 2010). Cross-sample entropy is similar to sample entropy but defined for a pair of time series. The analysis confirmed the synchronized dynamics of neuromagnetic activity between SI and PPC, robustly expressed across subjects. As no regions of interest were defined *a priori*, the map of pattern synchronizations between activated regions emerged automatically on the noisy background. The patterns contained in PPC regions were found to be well coordinated with those in SI, not necessarily being phase locked.

In spite of some progress made in understanding the SSSR, the question on the different stages of the development of the SSSR requires a further exploration. For example, in studies on the auditory steady-state response (ASSR) as a result to a periodically acoustic signal, it has been demonstrated that the development of the ASSR to amplitude-modulated sound occurs in cortical regions at slower modulation rates (<60 Hz) and in brainstem at faster modulation rates (>60 Hz) (Dimitrijevic and Ross, 2008). Despite analogous functional implications about the spatial characteristics of the SSSR, a better understanding of the somatosensory processing is expected.

Establishing a fully data-driven pipeline, this study explored the transmission of information between neuromagnetic sources in a functional network activated by vibrotactile stimulation of the right index finger. First, whole-brain neural activity representing the somatosensory steady-state response was reconstructed with a beamformer technique. Then, the information exchange was quantified between the primary somatosensory area SI and the rest of the brain. The volumetric map of the asymmetry in information transfer with respect to SI was created. We identified several brain areas wherein interactions with area SI were consistent across subjects. Specifically, the sources were localized contralaterally to the stimuli in the secondary somatosensory area SII, the thalamus, the motor cortex, and ipsilaterally to the stimuli in the cerebellum.

Thalamus sends and receives signals to and from cortical as well as to and from sub-cortical areas within the local and large-scale thalamo-cortical circuitry (Ribary et al., 2014). Five out of about 50 nuclei of the thalamus act as relays, receiving inputs from sensory peripheral receptors and sending the information to the primary sensory cortex for sophisticated processing(Ward, 2013). The relay nuclei which innervate the primary sensory projection areas, can be called the first-order relay nuclei. The other 45 higher-order thalamic nuclei participate in complex cortical and sub-cortical networks, relaying information between cortical and subcortical areas without primary sensory inputs (Sherman, 2012).

MEG has a limited spatial resolution and is not able to distinguish between first-order and higher-order thalamic nuclei. Directed coupling between area SI and thalamus, as found in our study, represent an averaged information transfer between these two brain regions. The imbalance in functional connectivity observed as SI → thalamus, would be consistent with a variance in the number and efficacy of the cortico-thalamic and thalamo-cortical projections, assuming similarities between the visual and somatosensory systems. It is estimated that there are 10-100 cortico-geniculate axons for every geniculo-cortical axon (Sherman and Koch, 1986). In cats, it was estimated that about 60% of the synaptic terminals in the lateral geniculate nucleus (LGN) are cortical in origin, where only about 10% come from retina (Montero, 1991).

As for anatomical connections between SI and SII, it was often found, at least in the mouse, when the cortical areas are mutually interconnected, they are also connected through a cortico-thalamo-cortical pathway arranged in parallel. The prevailing belief was originally based on an assumption that information is transmitted through direct cortico-cortical pathways (Kaas, 1987). For instance, in the visual system, a corollary of such a postulate would be that the information sent from the lateral geniculate nucleus to the primary visual cortex, remains primarily in the cortex when it is transferred up the higher levels of the cortical hierarchy (Olshausen et al., 1993). However, other studies suggest that after the first transmission of sensory information to the cortex from the periphery, much of information exchange between cortical areas is performed by the higher-order thalamic relays through the cortico-thalamic-cortical routes (Sherman, 2001; Guillery and Sherman, 2002; Sherman and Guillery, 2002). For example, using optical imaging, Theyel et al. (2009) studied the cortical hierarchy in slices of the mouse brain, involving the thalamus, primary and secondary somatosensory areas. Their results indicate that direct cortico-cortical projections from SI are not necessary to activate SII. On the contrary, it was a cortico-thalamic-cortical circuit starting in area SI that strongly activated area SII.

Note that in our study, we report only the dominant part of bi-directional communication between the cortical areas. We found that more information was transferred from area SI to area SII, than vice versa. Physiologically, it may seem to be a trivial result, as it is consistent with both hypotheses on information processing in cortical hierarchy. Methodologically, however, the analysis was performed in a fully data-driven manner. It is interesting to report that the pathway SI → SII was identified among all the possible connections between an activated source in area SI and all other sources defined by the nodes of the grid used to reconstruct the whole-head neuromagnetic activity.

We also identified connections leading from area SI to the motor cortex and the cerebellum. A number of studies has shown that in the rat SI can project to important motor control centers (Petrof et al., 2015) as well as to cerebellum (Bower et al., 1981). Both areas showed activation in response to electrically stimulated SI (Brown and Bower, 2002). Specifically, SI projected to the cerebellar granule cell patches either contralaterally or ipsilaterally, depending on the laterality of the peripheral projections of those patches. It was proposed that the cerebellum can be responsible for coordinating the acquisition of sensory data, which is further process by the nervous system, at least in the rats (Bower and Woolston, 1983; Morissette and Bower, 1996). In the human brain, a number of functional MRI (fMRI) studies reported ipsilateral activation of the cerebellar sources in reaction to tactile stimuli. For example, passive movement with the tactile system activated the primary supplementary motor and pre-motor cortex, as well as SI and SII contralaterally and the cerebellum ipsilaterally (Yang et al., 2011). Similar results were found in another fMRI study involving stroking and touching tasks (Zaman et al., 2001).

In general, our results thus demonstrate that exploring the asymmetries in information transfer in the propagation of a steady-state response with information-theoretic tools is a powerful method for describing information processing in a data-driven manner, and is able to reveal coordinated activity hidden from conventional analysis of functional connectivity. For central processing of sensory information, our results were found to be consistent with previously reported empirical evidence on coupling between primary somato-sensory area SI and secondary area SII, thalamus, motor cortex, and cerebellum.

## Limitations of the Study

In our study, we quantify only net transfer of information, not considering information flows in both directions separately. The rational for this is that in the models based on coupled oscillators, unidirectional coupling may lead to spurious detection of a bidirectional coupling (Bezruchko and Smirnov, 2010). In other words, in general, we cannot distinguish two situations: a unidirectional coupling or bidirectionally coupled systems with a weak coupling in one direction and a strong coupling in the other. Also note that we quantify asymmetry in information transfer, meaning that equal information flows in both directions will cancel each other. This would potentially explain why we do not observe activation in some areas like posterior parietal cortex (PPC) with effective connectivity. Specifically, in our previous study (Vakorin et al., 2010), we identified a connection between SI and bilateral PPC, using a measure called cross-sample entropy, which represents a non-linear method for estimating functional connectivity. This would imply that neurodynamics at SI and PPC are essentially similar, and therefore net information transfer between them is close to zero. In the current study, we quantify a dis-balance in information transfer, reporting connections with a high asymmetry.

We limited our analysis to the canonical beta frequency band, which was associated with the stimulus frequency. We did not divide the whole beta band into sub-bands. A number of studies have indicated that the directionality of coupling for spectrally resolved statistics can be sensitive to phase delays (or advances) between the signals in the presence of strong phase synchronization (Vakorin et al., 2013). We used a canonical beta frequency band, which on the one hand, can be associated with the stimulus frequency, and on the other hand, is relatively wide to avoid spurious effects related to phase synchronization.

We also acknowledge that our study may have been limited by a relatively small sample size (*n* = 10), although, as described in section Results, effect size for activation clusters was strong, based on Hedgesg statistic (Hedges, 1981).

## Author Contributions

VV, BR, and AM: Concept and design of the study; VV and BR: Data acquisition and analysis; VV, BR, SD, UR, and AM: Drafting the manuscript; VV: Preparing the figures.

### Conflict of Interest Statement

The authors declare that the research was conducted in the absence of any commercial or financial relationships that could be construed as a potential conflict of interest.
